# Employees and the National Insurance Act

**Published:** 1912-08-17

**Authors:** 


### NATIONAL INSURANCE ACT DEPARTMENT.

### EMPLOYEES AND THE NATIONAL INSURANCE ACT.

### XXIV.—The Financial Provisions.

One day last week a leading London morning newspaper contained an article on the National Insurance Act under the heading "Is the Act Financially Sound?" This is, of course, a most important question and one that demands an answer in the affirmative if the scheme is to be a permanent success. It must be admitted that the test of success or of failure depends upon the validity of the financial provisions and on the practical manner in which the principles are applied.

#### A Criticism Answered.

The article, then, proceeded to criticise the basis of the Act, and the conclusion at which the writer arrived was that the principles are unsound whilst the details of working are complex and costly. If this conclusion were correct those who are affected by the Act, whether as employers or employed, or even as taxpayers, would have just cause for uneasiness, and we have no doubt that such a feeling came to many, amongst them perhaps some of our readers, who read the article in question. It is because we hold, on grounds presently explained, that the Act is sound in the matter of its financial principles, and that, speaking generally, the details of the scheme are well designed, that we take this opportunity of allaying any fears that the article, or others like it, may have left.

The Act's imperfections are many and troublesome to a degree to those who have had to devote close attention to its provisions. We do not intend to furnish a formal answer, point by point, to the particular criticism referred to, but merely to make use of certain statements contained in the article in order to emphasise the fallacious lines of reasoning that may so easily be taken when the subject-matter is outside the usual range of everyday acquaintance. It has also to be borne in mind that, in spite of the writer's statement above quoted, political bias has much to do with the point of view taken when examining the principles underlying an Act of Parliament.

#### The Financial Soundness of the Act Explained.

The occasion is opportune, for, although we have necessarily referred to the relation of benefits to contributions and to reserves, we have not explained in detail the provisions of the Act which specially refer to the financial soundness of the scheme. To appreciate the matter fully, a certain amount of technical knowledge of an actuarial nature is necessary; but it can be popularly explained. The central fact of sickness insurance is the increase in human liability to disease and disablement in advancing years. Every insurance scheme must therefore reckon with the fact that members who have passed middle age will draw on the funds of their society much more heavily than the young. There are two alternative ways in which these heavy calls may be met, and societies have been formed on each basis, and thus each method has been practically tested.

#### The Dividing-out System.

The first of these methods may be described as the dividing-out system, and societies that have adopted this plan are usually called "Dividing Societies." In this class of society the benefits and contributions are the same for all members, whatever their age, and the payments are so arranged as to be substantially equal in total to the benefits paid to the members as a whole. At the end of each year there is a dividing-out amongst the members of any surplus funds, and this feature is frequently, we understand, one of the chief attractions of this class of society. It is usual to retain a small balance in hand to enable the treasurer to meet the claims for benefit that may accrue immediately after an annual division and to furnish a slight inducement for the continuation of membership, as well as providing a fund for use in emergency in case of unusual stress of claims. Apart from this small balance carried forward from year to year no reserves are accumulated. Each year's payments must be provided by the receipts for that year. It will immediately be seen that as the young man does not receive so much in benefits as the old man whilst both pay the same contribution, the system really resolves itself into this, that the young man pays for the old man of a previous generation. As time goes on, the demands made by the young man upon the funds increase with increasing age, until he is himself a member of the generation passing away, drawing heavily upon his society, and himself a burden upon the generation that succeeds him. By this means he draws in old age from the contributions of the next generation what he contributed in earlier life to the generation preceding him. Life assurance societies have been founded on the same principle, which in this connection is known as the "assessment plan."

The continued success of a society based on this plan, whether for sickness insurance or life insurance, depends upon the influx of new members. Having no reserve its liability to its old members can only be discharged so long as it can secure new members whose claims are less than their contributions. As soon as the influx of new members declines and the average age of the members increases the charge per member for the benefits increases, with the result that new members become exceedingly difficult, if not impossible, to obtain, whilst the younger members secede, until at last the society has to be wound up without making provision for the old members. The latter are then in the unfortunate position of having contributed to the claims of a previous generation without deriving a corresponding benefit from the one succeeding. This is the radical defect in the dividing-out plan as applied to private societies, and although in a National scheme, such as that which is at present in force in Germany, there is no possibility of failure of new entrants, the scheme is nevertheless economically unsound, because it ignores the fact that each generation should provide for its own needs instead of for those of a former generation.

### The Accumulation Plan.

The alternative plan may be called the "accumulative system," and this is the basis that has been adopted in the National Insurance Act. It is also the basis of all the great Friendly Societies and Life Insurance Institutions. By this system the young person does not pay for the claims of the old persons of a former generation. It is true that the contributions are more in early life than are required to meet the claims of that period, but the surplus of contributions over payments is carefully reserved and invested at interest to provide for the time when benefits will exceed contributions. Thus the new entrant pays directly for his own old age and for that of persons of his own age who are similarly insured. In a private insurance society founded on correct principles, it is essential that contributions should increase with the age at entry into insurance. This point is clearly recognised by the writer of the article to which we alluded, and he is at a loss to understand how the Act can be financially sound, when it so far departs from this principle as to require a contribution at a "flat rate" for the same benefit at all ages from 16 to 50 in the case of employed persons, and from 16 to 45 in the case of voluntary contributors entering insurance before January 15 next. His conclusion is that the "flat rate" must be unjust to the young insured person by making him pay for the old person. A careful examination will show that this is incorrect, though it has to be admitted that the young person does not derive so much advantage from the State grant towards the benefits as the older person.

The actuaries advising the Government were asked to calculate the contribution required at the age of sixteen to cover seven-ninths of the benefits and the cost of administrating them. This proportion only held in the case of men; for women the proportion was three-quarters, but exactly the same principle is involved in both cases, and it is only necessary to deal with the case of men for the purpose of this explanation. On the most appropriate tables available the actuaries calculated that the weekly contribution required to provide the full benefits was 7d., and that in consequence the weekly contribution for seven-ninths of the benefits was forty-nine-ninths of a penny, or $5\frac{4}{9}$d.

The balance of two-ninths of all benefits and two-ninths of the cost of management expenses is borne by the Government out of moneys provided by Parliament. Now the "accumulation plan," taken in conjunction with a flat rate of contribution, requires a reserve to be held in respect of everyone insured over the age of sixteen, and the great handicap under which the National scheme had to be launched was this, that by taking into insurance above the age of sixteen something like ten million persons who had not previously been insured under the scheme, there was no reserve laid by as there should have been. The estimated amount of the reserve required was about £66,000,000, and this sum has to be treated as an investment at interest in order that the scheme may be financially sound. It was absolutely essential that this sum should be provided, but to raise a loan of this magnitude would have entailed a dislocation of finance that would have been highly undesirable, and the charge for interest alone would have been a severe strain on the taxpayer. The method by which the deficiency is being liquidated is most ingenious.

### Liquidation of the Deficiency.

The cost of the full benefits is represented by a weekly contribution of 7d., payable as from the age of sixteen, but as the State is providing two-ninths of the benefit the actual weekly contribution required at that age is $5\frac{4}{9}$d. as already explained. The difference between these sums is $1\frac{5}{9}$d., and this sum is being deducted from each weekly contribution of 7d. and applied towards the liquidation of the deficiency. Here, then, is the position of a young man entering insurance at the age of sixteen. A weekly contribution of 7d. is being charged for benefits worth 7d. a week, thus he is getting the full value for the contributions paid by himself and by his employer on his behalf. By means of the State grant towards the benefits the contributions could have been reduced by $1\frac{5}{9}$d. a week if it had not been that others older than himself were to be compulsorily insured. What the State has given in the proportion of benefits it has taken from the contribution to liquidate the deficiency. This is, however, only a temporary arrangement, as it is estimated that in eighteen years or thereabouts the whole sum will have been written off together with its interest accumulations. When this time arrives it will be possible to increase the benefits, and provision has been made in the Act for the revision of the benefits accordingly. In the meantime the persons who obtain the advantage of the State grant are those who enter insurance at ages above sixteen, for while the same deduction of $1\frac{5}{9}$d. is made from each contribution the State grant of two-ninths of the benefits becomes increasingly valuable with the increase in age.

No private insurance company or friendly society could have launched a scheme on these lines, simply because no private concern would have the funds to supply a proportion of the benefits apart from the contributions of its own members. It is not possible, therefore, to base a valid criticism of the financial soundness of the measure on the undoubted fact that no private institution could have launched a similar scheme.

---

**NOTE.—Questions and Correspondence on all doubtful points are invited, and should be addressed to the Editor, THE HOSPITAL, 28 Southampton Street, Strand, W.C., and marked "Insurance."**

